# Sensory Denervation Delays Burn Wound Healing in Experimental Second- and Third-Degree Burns: A Histopathological Rat Study

**DOI:** 10.3390/ebj7030039

**Published:** 2026-07-15

**Authors:** Ugur Horoz, Hülda Rifat Ozakpinar, Emre Inozu, Ergin Seven, Avni Tolga Eryilmaz, Haldun Umudum, Umut Dogu Akturk, Ali Teoman Tellioglu

**Affiliations:** 1 Private, Plastic Reconstructive and Aesthetic Surgery Clinic, Istanbul 34365, Türkiye; 2 Plastic Reconstructive and Aesthetic Surgery Department, Lokman Hekim University, Ankara 06510, Türkiye; rft1969@gmail.com; 3 Private, Plastic Reconstructive and Aesthetic Surgery Clinic, Ankara 06510, Türkiye; dremre78@gmail.com (E.I.); erginseven@gmail.com (E.S.); mdtolgaer@yahoo.com (A.T.E.); aliteoman.tellioglu@gmail.com (A.T.T.); 4 Pathology Department, Ufuk Unıversity, Ankara 06520, Türkiye; humudum@yahoo.com; 5 Neurosurgery Department, Lokman Hekim University, Istanbul 34912, Türkiye; umutdoguakturk@hotmail.com

**Keywords:** burn injury, sensory denervation, wound healing, epithelialization, fibroblast proliferation, collagen deposition, rat model

## Abstract

**Highlights:**

**What are the main findings?**

- Sensory denervation significantly delayed burn wound healing, particularly thermal burn injuries.
- Denervated wounds demonstrated reduced epithelialization, impaired fibroblast proliferation and diminished collagen deposition compared with innervated wounds.

**What are the implications of the main findings?**

- Sensory innervation plays a critical role in coordinating tissue regeneration and extracellular matrix remodeling following burn injury.
- Preservation of neural integrity may represent a potential therapeutic target for improving burn wound healing and regenerative outcomes.

**Abstract:**

**Background**: Burn wound healing is a complex biological process requiring coordinated interactions among inflammatory cells, keratinocytes, fibroblasts, endothelial cells, extracellular matrix components, and neural structures. Increasing evidence suggests that sensory nerves actively regulate tissue repair through modulation of inflammation, angiogenesis, epithelial regeneration, and tissue remodeling. However, the role of sensory innervation in burn wound healing remains incompletely understood. The aim of this study was to investigate the effects of sensory denervation on healing of experimental second- and third-degree burns in rats. **Methods**: Thirty-two adult male Wistar albino rats were randomly allocated into four groups: denervated second-degree burns (Group 1), denervated third-degree burns (Group 2), innervated second-degree burns (Group 3), and innervated third-degree burns (Group 4). Bilateral T5–T7 sensory denervation was performed microsurgically in denervated groups. Standardized contact burns were subsequently created. Histopathological assessment of inflammation, neovascularization, epithelialization, fibroblast proliferation, and collagen deposition was performed on postoperative days 3, 7, 14, and 21. **Results**: Sensory denervation adversely affected multiple parameters of burn wound healing. Delayed epithelialization, impaired fibroblast proliferation, and reduced collagen deposition were most pronounced in denervated third-degree burns. Significant differences were identified between denervated and innervated wounds, particularly during proliferative and remodeling phases of healing. **Conclusions**: Sensory denervation significantly delays burn wound healing, particularly following third-degree thermal injury. Intact sensory innervation appears essential for successful epithelial regeneration, fibroblast activation, and extracellular matrix remodeling.

## 1. Introduction

Burn injuries remain one of the leading causes of morbidity worldwide and continue to represent a major challenge in reconstructive and regenerative medicine. Despite considerable advances in burn care, delayed wound healing, hypertrophic scarring, and functional impairment remain common complications, particularly following deep dermal and full-thickness burns. Successful burn wound healing requires a highly coordinated interaction among inflammatory cells, keratinocytes, fibroblasts, endothelial cells, extracellular matrix components, and the peripheral nervous system [1,2,3].

Increasing evidence suggests that peripheral sensory nerves actively regulate cutaneous homeostasis and wound repair beyond their conventional role in nociception. Sensory nerve fibers release numerous neuropeptides and trophic mediators that influence keratinocyte migration, fibroblast proliferation, angiogenesis, immune cell recruitment, and extracellular matrix remodeling. Consequently, disruption of neural integrity has been associated with impaired tissue regeneration and delayed wound healing in both experimental and clinical settings. Experimental studies have demonstrated that denervation alters inflammatory responses, delays wound contraction, reduces collagen synthesis, and impairs re-epithelialization. Likewise, chronic wounds in patients with peripheral neuropathy or spinal cord injury further emphasize the importance of intact neural signaling during tissue repair [4,5,6,7,8,9,10].

Although the relationship between denervation and impaired wound healing has previously been reported, relatively few studies have specifically investigated sensory denervation in standardized thermal burn models using serial histopathological evaluation throughout the different phases of burn wound healing. Most available studies have focused on excisional wounds, mixed wound models, or clinical observations rather than controlled experimental burn injury [6,7,8,9,11,12,13,14,15,16].

Burn wound healing progresses through overlapping inflammatory, proliferative, and remodeling phases, each characterized by distinct cellular events. The effects of sensory denervation may vary throughout these biological stages, influencing inflammatory cell recruitment during the early phase and fibroblast activation, collagen deposition, and epithelial regeneration during later stages. Therefore, evaluating these parameters at multiple time points may provide a more comprehensive understanding of the contribution of sensory innervation to burn wound repair.

The present experimental study aimed to investigate the effects of sensory denervation on second- and third-degree contact burn healing in a standardized rat model using serial histopathological assessment performed on postoperative days 3, 7, 14, and 21. We hypothesized that sensory denervation would impair burn wound healing by reducing epithelialization, fibroblast proliferation, and collagen deposition, particularly in deep thermal injuries.

## 2. Materials and Methods

### 2.1. Experimental Animals

Thirty-two adult male Wistar albino rats weighing 250–300 g were included in this experimental study. Animals were housed individually under standard laboratory conditions with controlled temperature (22 ± 2 °C), relative humidity (55 ± 10%), and a 12 h light/dark cycle. Standard laboratory chow and water were provided ad libitum throughout the study. All experimental procedures were approved by the Institutional Animal Care and Use Committee and were performed in accordance with the National Institutes of Health Guide for the Care and Use of Laboratory Animals.

### 2.2. Experimental Design

Animals were randomly assigned into four experimental groups (*n* = 8 per group):

Group 1: Sensory denervation + second-degree burn.

Group 2: Sensory denervation + third-degree burn.

Group 3: Innervated second-degree burn.

Group 4: Innervated third-degree burn.

### 2.3. Surgical Denervation

Animals assigned to the denervated groups underwent bilateral sensory denervation of the dorsal T5–T7 dermatomes under general anesthesia. Following a midline dorsal skin incision, the paraspinal muscles were gently retracted to expose the dorsal sensory nerve branches. The bilateral sensory branches supplying the T5–T7 dermatomes were carefully isolated from surrounding tissues and transected under magnification while preserving adjacent vascular structures. The surgical incision was subsequently closed in anatomical layers.

**Figure 1:**
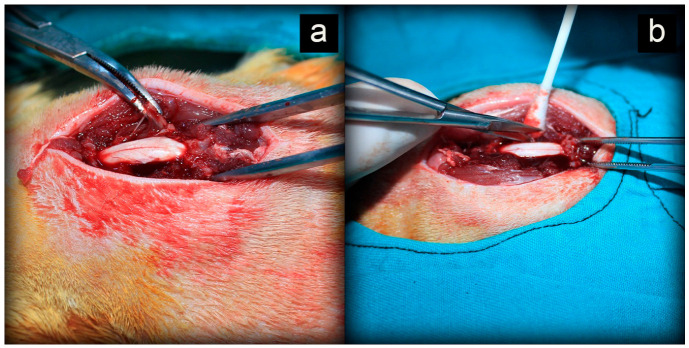
Surgical sensory denervation procedure. (**a**) Exposure of the paraspinal region following skin incision and identification of the dorsal sensory nerve branches supplying the T5–T7 dermatomes. (**b**) Isolation and bilateral transection of the sensory nerve branches to achieve experimental denervation.

A recovery period of three days was allowed before burn induction to ensure complete interruption of sensory innervation while minimizing the influence of acute surgical inflammation on subsequent burn wound assessment (Figure 1).

### 2.4. Burn Model

Following anesthesia and dorsal hair removal, standardized bilateral contact burns were created using a custom-designed heated metal plate measuring 2 × 2 cm.

In the denervated groups, burns were created within the previously denervated T5–T7 sensory territory while remaining spatially separate from the midline surgical incision used for denervation. Consequently, the burn wounds and the surgical incision represented anatomically distinct wound sites and were evaluated independently throughout the study.

In the innervated groups, no surgical incision or denervation procedure was performed. Following identical anesthesia and skin preparation, standardized contact burns were directly created on intact dorsal skin over the corresponding T5–T7 dermatomal region using the same burn protocol.

Second-degree burns were produced using a metal plate heated to 100 °C applied for 45 s, whereas third-degree burns were induced using a metal plate heated to 120 °C applied for 60 s.

### 2.5. Histopathological Evaluation

Histopathological specimens were obtained from the peripheral margin of each burn wound on postoperative days 3, 7, 14, and 21. Tissue sampling was performed in a standardized manner that minimized interference with the remaining wound area, allowing animals to survive until completion of the 21-day follow-up period.

Specimens were fixed in 10% buffered formalin, embedded in paraffin, sectioned at 4 µm thickness, and stained with hematoxylin–eosin (H & E). Histopathological evaluation was performed by an experienced pathologist blinded to group allocation using light microscopy.

The following parameters were assessed using a semi-quantitative scoring system:

- Inflammation: 0 = absent; 1 = mild/moderate; 2 = severe.
- Fibroblast proliferation: 0 = absent; 1 = partial; 2 = marked.
- Collagen deposition: 0 = absent; 1 = partial; 2 = prominent.
- Neovascularization: 1 = absent/minimal; 2 = prominent.
- Re-epithelialization: 0 = absent; 1 = partial; 2 = complete.

### 2.6. Statistical Analysis

Statistical analyses were performed using MedCalc Statistical Software, version 12.7.7 (MedCalc Software bvba, Ostend, Belgium; http://www.medcalc.org (accessed on 31 May 2026); 2013). Categorical variables were presented as numbers and percentages and compared using the chi-square test or Fisher’s exact test, as appropriate. A two-sided *p*-value < 0.05 was considered statistically significant.

## 3. Results

### 3.1. Macroscopic Findings

All animals survived throughout the 21-day experimental period. Following thermal injury, all burn wounds developed characteristic eschar formation followed by progressive contraction and epithelial regeneration. Macroscopic evaluation demonstrated clear differences in wound healing between denervated and innervated animals.

Healing progressed more rapidly in second-degree burns than in third-degree burns regardless of innervation status. However, sensory denervation delayed wound contraction and epithelial coverage in both burn depths. These differences became increasingly apparent during the later stages of wound healing and were most pronounced on postoperative day 21.

**Figure 2:**
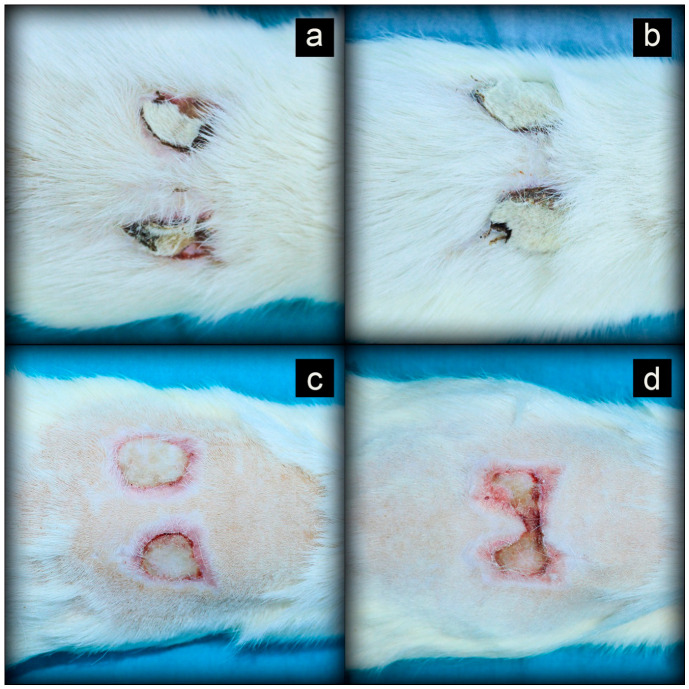
Representative macroscopic appearance of burn wounds on postoperative day 21. (**a**) Group 1: Denervated second-degree burn. (**b**) Group 2: Denervated third-degree burn. (**c**) Group 3: Innervated second-degree burn. (**d**) Group 4: Innervated third-degree burn. Delayed wound closure and persistent tissue loss are particularly evident in denervated third-degree burn wounds.

Among all experimental groups, Group 2 (denervated third-degree burns) exhibited the poorest macroscopic healing, with persistent tissue defects, incomplete wound closure, and delayed contraction. In contrast, Group 3 (innervated second-degree burns) demonstrated almost complete wound closure with minimal residual scar formation by postoperative day 21. Representative macroscopic findings are presented in Figure 2.

### 3.2. Inflammatory Response

Inflammatory cell infiltration increased during the early inflammatory phase in all groups.

**Figure 3:**
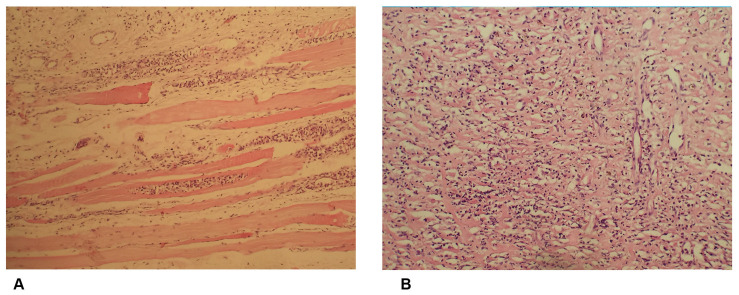
(**A**) Postoperative day 3: Dense mixed inflammatory cell infiltration is observed between the skeletal muscle bundles, representing the acute inflammatory phase following thermal injury. (**B**) Postoperative day 21: Dense inflammatory infiltrates composed predominantly of macrophages and mononuclear lymphocytes are observed between collagen bundles, consistent with the late inflammatory/remodeling phase of wound healing.

On postoperative day 3, inflammatory activity was significantly greater in the innervated groups (Groups 3 and 4) than in the denervated groups (Groups 1 and 2) (100% vs. 53.3%, *p* = 0.007). This finding suggests that intact sensory innervation contributes to an early inflammatory response following thermal injury. Figure 3 shows representative histopathological findings demonstrating inflammatory cell infiltration during burn wound healing (Hematoxylin–Eosin staining, ×20).

By postoperative days 14 and 21, inflammatory activity had substantially decreased in all groups, and no statistically significant differences were observed between denervated and innervated animals, indicating resolution of the acute inflammatory phase (Table 1).

### 3.3. Neovascularization

Neovascularization progressively increased during the proliferative phase of wound healing.

On postoperative day 14, significant differences were identified between Group 1 and Group 3. Neovascularization was present in 71.4% of specimens in Group 1 but was absent in Group 3 (*p* = 0.02). Likewise, all specimens in Group 4 demonstrated neovascularization, whereas none of the specimens in Group 3 showed vascular proliferation (*p* < 0.01).

By postoperative day 21, neovascularization had become well established in all groups, and no statistically significant intergroup differences remained (Table 2). These findings suggest that sensory denervation did not permanently impair angiogenesis but rather influenced its temporal progression during wound healing.

### 3.4. Epithelialization

Re-epithelialization represented the histopathological parameter most strongly affected by sensory denervation.

In second-degree burns, epithelial regeneration was considerably better in the innervated group than in the denervated group throughout follow-up. Although partial epithelial regeneration was observed in Group 1, Group 3 exhibited earlier and more complete epithelial coverage during the later stages of healing.

The effect of denervation became even more pronounced in third-degree burns. On postoperative day 21, complete epithelialization was absent in Group 2, whereas epithelial regeneration was observed in 62.5% of specimens in Group 4.

Overall epithelialization rates on postoperative day 21 were:

- Group 1: 28.6%.
- Group 2: 0%.
- Group 3: 87.5%.
- Group 4: 62.5%.

Group 2 demonstrated significantly lower epithelialization than both Group 3 (*p* < 0.01) and Group 4 (*p* = 0.04). Representative histopathological findings are shown in Figure 3, while quantitative data are summarized in Table 3.

**Figure 4:**
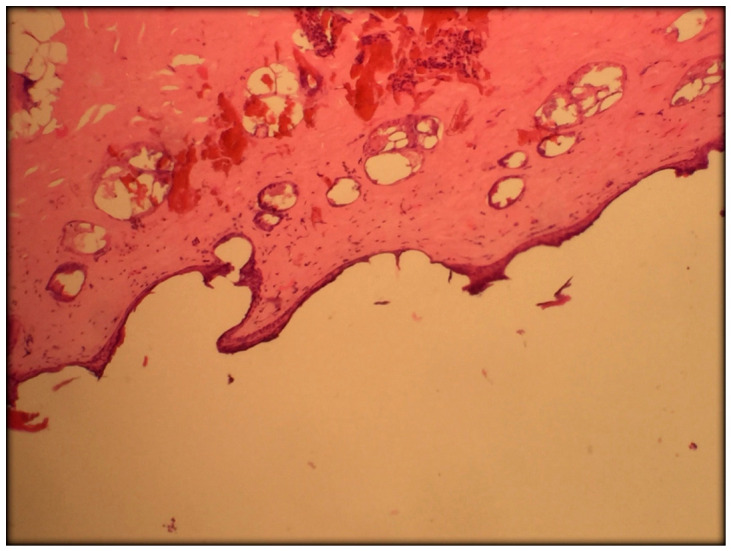
Representative histopathological findings demonstrating re-epithelialization on postoperative day 21 (Hematoxylin–Eosin staining, ×4). Re-epithelialization was markedly reduced in denervated wounds, particularly in Group 2.

Overall, epithelial regeneration was markedly delayed in denervated third-degree burn wounds (Figure 4).

### 3.5. Collagen Deposition

Collagen deposition progressively increased throughout the remodeling phase of healing.

Although collagen formation was observed in all groups by postoperative day 21, marked differences were evident between burn depths and innervation status.

In second-degree burns, collagen deposition remained relatively preserved regardless of denervation. Conversely, denervated third-degree burns demonstrated substantially impaired extracellular matrix formation.

On postoperative day 21, collagen deposition was observed in:

- Group 1: 100%.
- Group 2: 28.6%.
- Group 3: 75.0%.
- Group 4: 100%.

Collagen deposition was significantly lower in Group 2 than in both Group 1 and Group 4 (*p* = 0.02), indicating that sensory denervation markedly impaired matrix remodeling following deep thermal injury.

### 3.6. Fibroblast Proliferation

Fibroblast proliferation differed significantly during the early proliferative phase of wound healing.

On postoperative day 3, fibroblast proliferation was observed in:

- Group 1: 75.0%.
- Group 2: 28.6%.
- Group 3: 62.5%.
- Group 4: 100%.

The lowest fibroblast activity was observed in Group 2, which demonstrated significantly reduced proliferation compared with Group 4 (*p* = 0.01).

In second-degree burns, fibroblast proliferation remained relatively preserved despite sensory denervation, whereas third-degree denervated wounds demonstrated a marked delay in fibroblast activation during the early proliferative phase.

**Figure 5:**
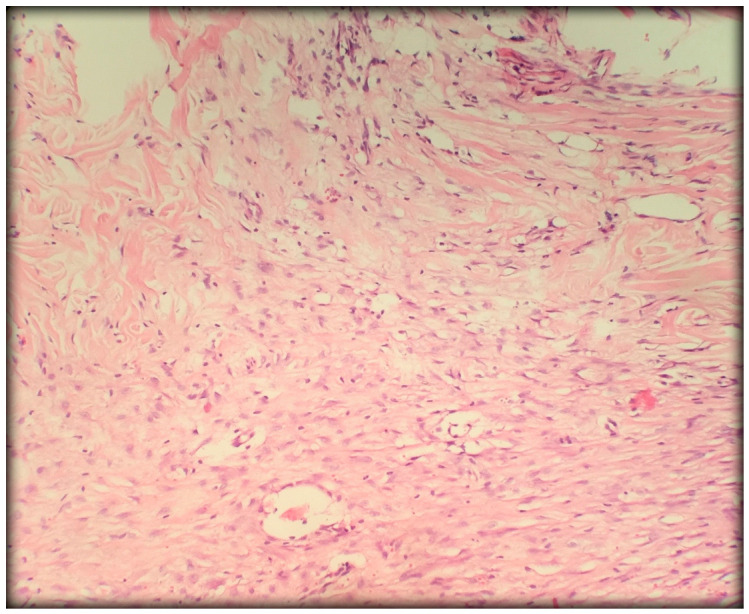
Representative histopathological findings demonstrating fibroblast proliferation on postoperative day 21 (Hematoxylin–Eosin staining, ×20). Increased fibroblast proliferation was observed in innervated wounds compared with denervated wounds.

By postoperative day 21, fibroblast proliferation had increased substantially in all experimental groups, and no statistically significant differences remained. Nevertheless, the delayed early fibroblast response observed in denervated third-degree burns likely contributed to the impaired collagen deposition and delayed wound healing observed during the remodeling phase. Representative histopathological findings are presented in Figure 5.

## 4. Discussion

The present study demonstrated that sensory denervation significantly impairs burn wound healing, particularly following third-degree thermal injury. The principal findings were delayed re-epithelialization, reduced fibroblast proliferation, and diminished collagen deposition in denervated wounds compared with innervated controls. Importantly, these effects varied throughout the different stages of wound healing, indicating that sensory innervation contributes not only to the initiation of tissue repair but also to the proliferative and remodeling phases of burn wound healing.

Although the relationship between denervation and impaired wound healing has previously been described, relatively few experimental studies have specifically evaluated standardized second- and third-degree burn wounds using serial histopathological assessment throughout the healing process. Most previous investigations have focused on excisional wounds or mixed wound models rather than standardized thermal injuries. Recent reviews have emphasized that neurocutaneous interactions remain incompletely understood in burn pathology and require further experimental investigation [17,18,19,20,21,22]. Thermal injury represents a unique pathological condition characterized by extensive coagulation necrosis, prolonged inflammation, and delayed tissue remodeling. Consequently, the biological consequences of sensory denervation may be substantially greater following burn injury than in conventional wound models. Our findings therefore extend previous observations by demonstrating that sensory denervation exerts stage-dependent effects throughout burn wound healing rather than influencing a single biological process.

The inflammatory response represents the initial phase of burn wound healing and plays a critical role in preparing the wound bed for subsequent tissue repair. Interestingly, inflammatory cell infiltration was significantly greater in the innervated groups during the early phase of healing but became comparable among all groups during later follow-up. These findings suggest that intact sensory innervation facilitates the early inflammatory response after burn injury, whereas denervation does not produce persistent inflammatory alterations. Similar temporal patterns have been reported in previous experimental studies demonstrating that delayed wound healing in denervated tissues primarily results from impaired progression to the proliferative phase rather than prolonged inflammation. This observation indicates that sensory nerves influence the quality and timing of the inflammatory response rather than its overall duration. These findings are consistent with current concepts of burn wound biology, in which controlled inflammatory signaling is essential for appropriate transition to the proliferative phase of healing [23,24].

Neovascularization increased progressively during the proliferative phase in all experimental groups. Although significant differences were identified on postoperative day 14, these differences were no longer present by day 21, suggesting that angiogenesis was delayed rather than permanently impaired by sensory denervation. Adequate vascularization is essential for oxygen delivery, nutrient transport, and fibroblast function during burn wound healing. Therefore, transient alterations in neovascularization may contribute to delayed progression toward tissue remodeling in denervated wounds. Adequate neurovascular communication has been recognized as one of the critical determinants of successful burn wound regeneration [23,24,25].

Among all evaluated parameters, re-epithelialization demonstrated the strongest association with sensory denervation. Complete absence of epithelialization in denervated third-degree burns on postoperative day 21 contrasted markedly with the substantial epithelial regeneration observed in innervated wounds. Similar observations were summarized by Jurjus et al., who concluded that disruption of peripheral sensory innervation delays epithelial regeneration and contributes to prolonged burn wound healing [22]. Even in second-degree burns, epithelial regeneration progressed more slowly following denervation despite preservation of viable dermal structures. These findings indicate that intact sensory innervation facilitates keratinocyte migration and epidermal regeneration regardless of burn depth, although the biological consequences become considerably more pronounced following severe thermal injury.

Our findings are consistent with those of Richards et al., Engin et al., Kim et al., Fukai et al., and Shu et al., who demonstrated delayed wound healing, impaired epidermal regeneration, and altered cellular responses following experimental denervation. The present study extends these observations by providing serial histopathological evaluation across multiple phases of standardized thermal burn healing rather than assessment at a single time point [22,23,24]. However, unlike previous investigations that primarily evaluated excisional wounds, the present study demonstrates similar pathological mechanisms within standardized burn wounds, thereby expanding current knowledge regarding neurocutaneous regulation of thermal injury repair. Furthermore, the comprehensive evaluation of multiple healing stages provides additional insight into the temporal effects of sensory denervation throughout burn wound healing.

Fibroblast proliferation was significantly reduced during the early proliferative phase, particularly in denervated third-degree burns. Fibroblasts play a central role in extracellular matrix synthesis, wound contraction, and growth factor production. Consequently, delayed fibroblast activation may explain the reduced collagen deposition observed during the remodeling phase. The absence of significant differences by postoperative day 21 suggests that sensory denervation delays fibroblast activation rather than completely preventing fibroblast recruitment. Emerging evidence suggests that peripheral nerve-derived mediators directly regulate fibroblast differentiation and extracellular matrix synthesis during tissue regeneration [23,25]. Nevertheless, this early delay appears sufficient to compromise subsequent extracellular matrix organization and overall wound maturation.

Collagen deposition represents one of the most important indicators of successful burn wound remodeling. In the present study, collagen formation remained relatively preserved in second-degree burns but was markedly reduced in denervated third-degree wounds. Deep burns require extensive extracellular matrix reconstruction because of widespread dermal destruction. Therefore, disruption of neural signaling may exert greater biological consequences when tissue regeneration depends predominantly on newly synthesized collagen. These findings support previous reports indicating that neural integrity contributes to dermal remodeling and scar maturation through regulation of fibroblast function and extracellular matrix turnover.

One of the most interesting observations of the present study was the different response between second- and third-degree burns. Although denervation negatively affected healing in both models, its impact was substantially greater following third-degree burns. Second-degree burns preserve portions of the dermis, hair follicles, and adnexal structures that may serve as reservoirs for epithelial regeneration. In contrast, third-degree burns require nearly complete tissue reconstruction, making successful healing more dependent upon coordinated neurovascular and cellular interactions. This difference probably explains the markedly impaired epithelialization and collagen deposition observed in denervated third-degree wounds.

From a clinical perspective, these findings may have important implications for patients with peripheral neuropathy, spinal cord injury, diabetes mellitus, or other neurological disorders associated with impaired sensory function. Recent advances in regenerative medicine have focused on preservation of neural integrity and stimulation of peripheral nerve regeneration as promising adjunctive strategies for improving burn wound healing and reducing pathological scar formation [22,25,26,27]. Delayed burn wound healing frequently observed in these patient populations may partly result from disruption of neurocutaneous signaling pathways. Preservation of neural integrity or therapeutic strategies aimed at enhancing peripheral nerve regeneration may therefore represent promising approaches for improving burn wound repair. Recent reviews, including that of Jurjus et al., have emphasized the growing importance of neurocutaneous interactions in burn healing and support further investigation into neuroregenerative therapies as adjuncts to conventional burn treatment.

The present study has several limitations. First, the number of experimental animals was relatively limited, although comparable with previous experimental burn studies. Second, molecular analyses of cytokines, neuropeptides, and growth factors were not performed. Third, immunohistochemical assessment of nerve regeneration was beyond the scope of the present investigation. Future studies integrating molecular, immunohistochemical, and functional analyses may further clarify the mechanisms through which sensory innervation regulates burn wound healing and may identify novel therapeutic targets for enhancing tissue regeneration following thermal injury. Integration of molecular analyses with histopathological findings may further clarify the complex neuroimmune interactions underlying burn wound healing [23,24,25,26].

Overall, the present findings provide additional experimental evidence that sensory innervation represents an essential biological component of successful burn wound healing. Rather than influencing a single phase of repair, sensory denervation appears to affect multiple sequential stages of tissue regeneration, ultimately resulting in delayed wound closure and impaired tissue remodeling.

## 5. Conclusions

Sensory denervation significantly impairs burn wound healing in experimental rat models, with its effects being particularly pronounced following third-degree thermal injury. Denervated wounds exhibited delayed re-epithelialization, reduced fibroblast proliferation, and diminished collagen deposition, indicating impaired progression through the proliferative and remodeling phases of wound healing. These findings underscore the essential role of intact sensory innervation in coordinating tissue regeneration and extracellular matrix remodeling after thermal injury. Similar mechanisms have recently been proposed in experimental burn healing models investigating neural regulation of dermal remodeling [22,24]. Furthermore, the stage-dependent histopathological alterations observed in this study suggest that preservation of peripheral sensory neural pathways may represent a promising therapeutic target for improving burn wound healing and developing future neuroregenerative strategies in burn care.

## Figures and Tables

**Table 1:** Histopathological findings according to study groups on postoperative day 3.

Parameter	Group 1 (%)	Group 2 (%)	Group 3 (%)	Group 4 (%)	*p*-Value
Inflammation	-	-	-	-	0.007 *
Fibroblast proliferation	75.0	28.6	62.5	100	0.01 †
Neovascularization	NS	NS	NS	NS	NS
Epithelialization	NS	NS	NS	NS	NS
Collagen deposition	NS	NS	NS	NS	NS

* Denervated groups vs. innervated groups; † Group 2 vs. Group 4; NS = not statistically significant.

**Table 2:** Histopathological findings according to study groups on postoperative day 14.

Parameter	Group 1 (%)	Group 2 (%)	Group 3 (%)	Group 4 (%)	*p*-Value
Neovascularization	71.4	-	0	100	0.02 */<0.01 †
Inflammation	NS	NS	NS	NS	NS
Fibroblast proliferation	NS	NS	NS	NS	NS
Epithelialization	NS	NS	NS	NS	NS
Collagen deposition	NS	NS	NS	NS	NS

* Group 1 vs. Group 3; † Group 4 vs. Group 3; NS = not statistically significant.

**Table 3:** Histopathological findings according to study groups on postoperative day 21.

Parameter	Group 1 (%)	Group 2 (%)	Group 3 (%)	Group 4 (%)	*p*-Value
Epithelialization	28.6	0	87.5	62.5	<0.01 */0.04 †
Collagen deposition	100	28.6	75.0	100	0.02 ‡/0.02 §
Fibroblast proliferation	NS	NS	NS	NS	NS
Inflammation	NS	NS	NS	NS	NS
Neovascularization	NS	NS	NS	NS	NS

* Group 2 vs. Group 3; † Group 2 vs. Group 4; ‡ Group 2 vs. Group 1; § Group 2 vs. Group 4; NS = not statistically significant.

## Data Availability

The data supporting the finding of this study are available from the corresponding author upon reasonable request. The datasets generated and analyzed during the current study are not publicly available but may be provided by the corresponding author for academic and research purposes.
